# The influence of organizational characteristics on employee solidarity in the long-term care sector

**DOI:** 10.1111/j.1365-2648.2012.06027.x

**Published:** 2013-03

**Authors:** Jane M Cramm, Mathilde MH Strating, Anna P Nieboer

**Affiliations:** 1 Institute of Health Policy & Management (iBMG), Erasmus UniversityRotterdam, The Netherlands; 2 Institute of Health Policy & Management (iBMG), Erasmus UniversityRotterdam, The Netherlands; 3 Institute of Health Policy & Management (iBMG), Erasmus UniversityRotterdam, The Netherlands

**Keywords:** communication, healthcare, leadership, management, nurse/nursing, organizational characteristics, solidarity

## Abstract

**Aim:**

This article is a report of a study that identifies organizational characteristics explaining employee solidarity in the long-term care sector.

**Background:**

Employee solidarity reportedly improves organizations’ effectiveness and efficiency. Although general research on solidarity in organizations is available, the impact of the organizational context on solidarity in long-term care settings is lacking.

**Design:**

Cross-sectional survey.

**Method:**

The study was carried out in Dutch long-term care. A total of 313 nurses, managers and other care professionals in 23 organizations were involved. Organizational characteristics studied were centralization, hierarchical culture, formal and informal exchange of information and leadership style. The study was carried out in 2009.

**Findings:**

All organizational characteristics significantly correlated with employee solidarity in the univariate analyses. In the multivariate analyses hierarchical culture, centralization, exchange of formal and informal information and transformational leadership appears to be important for solidarity among nurses, managers and other professionals in long-term care organizations, but not transactional and passive leadership styles.

**Conclusion:**

The study increased our knowledge of solidarity among nurses, managers and other professionals in the long-term care settings. Organizational characteristics that enhance solidarity are high levels of formal and informal information exchange, less hierarchical authority, decentralization and transformational leadership styles.

## Introduction

Employees of long-term care organizations are persistently pressured in their practice environments to meet competing demands within turbulent healthcare systems (Stone *et al.* 2003, Marck 2004, 2006, Shannon & French 2005, Johnson *et al.* 2006). They are witnessing a call from politicians, health policy analysts and scholars for improved efficiency and effectiveness. This gives nurses and other employees the challenging task of improving efficiency and effectiveness together (Appelbaum & Batt 1994), but some might be tempted to lean back and hitchhike on the work of others (Kerr 1983, Organ 1988). Cooperative, solidary behaviour is seen as one of the most important success factors in organizations (Wickens 1995) and understanding the factors influencing it in long-term care organizations is thus crucial. Following Katz (1964), dependence on voluntary participation and willingness to cooperate is interpreted herein as solidarity in nurses’ and other employees’ behaviour. Employee solidarity concerns employee behaviour that has an overall positive effect on the functioning of the organization and that cannot be enforced by the employment contract (Organ & Lingl 1995). Solidary behaviour between nurses, managers and other professionals occurs if employees in organizations contribute to the success of the team or organization, are prepared to help others in need, resist the temptation to let other members do most of the work, share responsibilities and are prepared to apologize for mistakes (Lindenberg 1998). Research has shown that it is related to employees’ resistance to organizational change (Torenvlied & Velner 1998) and short-term absenteeism (Sanders & Hoekstra 1998, Sanders 2004).

The notion of solidarity among nurses, managers and others in long-term care organizations is relatively new. In these organizations, employees work interdependently to deliver care. The nature of work in health care is characterized by increasing levels of interdependence (Gittell *et al.* 2000, Kaissi *et al.* 2003). Involvement of various nurses in healthcare delivery does not guarantee coordinated teamwork (Pearson 1983). Unfortunately, nurses, managers and other professionals in long-term care settings do not always work well together, which can negatively affect the quality of patient care and services (Kvarnstrom 2008). It is important to realize that nurses in long-term care settings have considerable autonomy, which is different from other industries. One problem is that they have to simultaneously manage the teamwork process and their individual tasks (Lingard *et al.* 2004). Such conditions may raise the potential for confusion, errors and delays (van Maanen & Barley 1984). Solidarity among nurses, managers and other professionals may encourage to value the contributions others make and consider the impact of their actions, reinforcing the inclination to act with regard for the overall work process.

Traditional research has studied solidarity among employees in conflict with management or in the enforcement of local work group norms (Roethlisberger & Dickson 1939, Seashore 1954, Blau 1955, 1964, Homans 1974). We are particularly interested in how organizational characteristics influence solidarity, i.e., cooperative behaviour characterized by reciprocity (Gouldner 1960, Hechter 1987) and the purpose of our study is to identify such characteristics in long-term care organizations in the Netherlands. We expect that differences in solidarity can in part be explained by them. In line with previous research, we define solidarity as behaving agreeably with other employees even when not convenient or formally described (Sanders *et al.* 2002).

Relationships in organizations are institutionally embedded, that is, they are influenced by the institutions that give the formal and informal rules and communication patterns that govern interaction between healthcare professionals (North 1990). Informal and formal exchanges of information in organizations complement the organization’s formal rules. For instance, employers often make use of social networks and informal social control to develop and maintain cooperative relations with and between employees (Flap *et al.* 1998). We, therefore, expect an organization’s informal and formal exchange of information patterns to be related to solidarity.

Empirical research shows that leadership style is highly effective in terms of commitment and motivation (Lowe *et al.* 1996). Although the relationship between leadership style and the supervisor–employee relationship has been studied (Podsakoff *et al.* 1990, 1993, 1996a, 1996b, Graen & Uhl-Bien 1995), research on the relationship between leadership styles and solidarity behaviour is rare, especially in long-term care organizations. Den Hartog *et al.* (1997) have studied three management styles: transformational, transactional and passive. ‘Transformational’ leaders ask followers to transcend their own self-interests for the good of the group, organization or society, and to consider long-term rather than momentary needs with respect to developing themselves. Transformational leaders upset the status quo and existing rule structures, replacing them with a ‘new order’ (Ferlie & Shortell 2001). Because transformational leaders ask employees to transcend self-interest for the good of the group, we expect them to be positively related to solidarity. ‘Transactional’ leaders build expectations by setting specific performance targets with their employees (Avolio & Bass 2002). Transactional leadership refers to ‘the exchange relationship between leaders and followers to meet their own self-interest’ (Bass 1990, p. 10). This type of leadership can be considered as effective as well, although the performance related to this leadership is lower than the one related to transformational leadership. ‘Passive’ leaders tend to react only after problems have become serious enough to take corrective action, and often avoid making decisions at all (Avolio & Bass 1999). While transactional leaders focus on own self-interests of employees, passive leaders are reactive in nature, and transformational leaders ask followers to transcend their own self-interests for the good of the group, we only expect a relationship between transformational leadership and solidarity.

Every organization has a culture that constitutes the expected, supported and accepted way of behaving. Cultural norms are mostly unwritten and tell employees how things ‘really are’. They influence everyone’s perception of the organization from the chief executive to the service worker. ‘Hierarchical’ and ‘centralized’ cultures by means of authority chains are vertical organizational structures (Taplin 1995). An organization’s hierarchy refers to how structured and inflexible its operation is and the extent to which authority is delegated to lower levels. We expect the inflexibility of a hierarchical culture and strong high-level authority to negatively affect solidarity among employees.

Employee solidarity reportedly improves organizations’ effectiveness and efficiency. Research shows that centralization, hierarchical culture, formal and informal exchange of information and leadership style are organizational characteristics affecting solidarity.

The impact of these organizational characteristics on solidarity in (long-term) healthcare settings is lacking. The aim of this study was to identify organizational characteristics explaining employee solidarity in the long-term care sector.

## The study

### Aim

The aim of the study was to identify organizational characteristics explaining employee solidarity in the long-term care sector.

### Design

This study used a cross-sectional design conducted in 2009.

### Participants

The study included 124 organizations participating between 2006–2009 in quality improvement programmes, which were part of a national Dutch programme called ‘Care for Better’. These quality improvements focused on specific topics namely: pressure ulcers, ill nutrition, prevention of sexual abuse, medication safety, fall prevention, problem behaviour, client autonomy and control, social participation, recovery-oriented care, somatic comorbidity of psychiatric clients and outreach care. In these organizations not all employees were involved in the quality improvement projects. This study was conducted among those employees not participating in the quality improvement projects. Of the124 organizations that received an open invitation to participate, 43 cooperated (35%) and 432 participants responded to the questionnaire. A total of 14 nursing homes (response rate 25%), 12 care organizations for disabled persons (response rate 36%) and 17 long-term mental healthcare organizations (response rate 50%) participated.

### Data collection

A package of questionnaires was sent to the contact person of each participating organization, which were distributed to potential respondents through their mail boxes or delivered personally at team meetings. Two weeks later we sent a reminder and copy of the questionnaire to non-respondents; 4 weeks later we sent a reminder only. No incentives in the form of money or gifts were offered. Because the questionnaires were indirectly distributed, the response rate for some organizations was low. Furthermore, some had several locations; being an external party made it difficult to check the actual distribution of questionnaires per location and respondent. We thus followed the recommendation of (van Mierlo *et al.* 2009) to include only organizations with response rates of at least 30%; 23 remained [average response rate 52·8% (sd 16·6)]: eight nursing homes (response rate 52·9%), six organizations for disabled persons (57·1%), nine long-term mental healthcare organizations (50·0%). The number of respondents per organization averaged 27·9 (sd 19·9).

### Ethical considerations

As this study included staff members only, we did not need approval for this study from an ethics committee. Informed consent was obtained from all participants. All personal identifiers were removed or disguised and so the person(s) described are not identifiable and cannot be identified through the details of the story.

### Data analysis

Descriptive analysis included the calculation of means and standard deviations. After calculating bivariate correlations, multiple regression analyses were used to predict solidarity in long-term care organizations.

### Validity and reliability/rigour

‘Solidarity’ was measured with ten items on a 5-point scale (see Appendix). Examples were ‘Everyone helps when something needs to be done’ and ‘In our team we help each other when there is a need for it’. Cronbach’s alpha of the instrument was 0·79. This scale is based on the theory on solidarity of Lindenberg (1998) that employees in organizations contribute to the success of the team or organization, are prepared to help others in need, resist the temptation to let other members do most of the work, share responsibilities and are prepared to apologize for mistakes. Koster *et al.* (2003) operationalized this theory and developed an instrument to assess solidarity among employees. An adjusted version of this instrument has been used in other settings to assess solidarity in schools (Kassenberg 2002), vacation communities (Philips 2005) and local exchange trading systems (Hoeben 1997).

‘Centralization’ was measured with four items on a 7-point scale (Dewar *et al.* 1980). Examples were ‘Little action can be taken here until a supervisor approves a decision’ and ‘Unit members need to ask their supervisor before they do almost anything’. Cronbach’s alpha was 0·78.

‘Hierarchical culture’ was assessed by asking employees’ perceptions of their organizational culture, in which respondents were asked to distribute 100 points across four sets of organizational statements across five areas according to descriptions that best fit their organization. One type of culture was hierarchical. Hierarchical culture was calculated by summing the points attributed to hierarchical culture on the five areas and divided by 5. The scores on hierarchical culture can vary between 0–100, higher scores indicating a more hierarchical culture (Cronbach’s alpha 0·71). Example of hierarchical culture is: “Management in this organization are typical rule-enforcers”.

‘Communication’ was measured with existing instruments for concepts such as potential and realized capacity, connectedness, knowledge creation and redundancy (Jansen *et al.* 2005, 2006, Lloria 2006). Subscales were formal internal exchange of information (six items) and informal internal exchange of information (three items). Examples were ‘Normally meetings are held to share knowledge, share ideas, and discuss issues related to work’; ‘In our organization there is ample opportunity for informal “hall talk’”; and ‘Employees of our unit regularly visit other organizations’. Cronbach’s alpha for the subscales were 0·75 (formal communication) and 0·74 (informal). ‘Leadership’ styles were assessed as transformational, transactional or passive according to a selection of items from the Multifactorial Leadership Questionnaire (Den Hartog *et al.* 1997, Avolio & Bass 1999). Transformational leadership was assessed with six items, transactional with five, and passive with four. All were rated on a 5-point scale (strongly disagree to strongly agree). Cronbach’s alpha for the subscales ranged from 0·67– 0·83.

## Results

### Sample characteristics

Respondents, whose median age was 42·1 (ranging from 16·7–63·6), were mostly women (75·5%). Almost half (41·9%) had completed tertiary education; 11·7% had a university degree. Most (70·8%) worked more than 30 hours per week and most (76·5%) had worked at the same organization for more than 3 years. A majority of the respondents (73·8%) consisted of medical professionals (mostly nurses) and 26·2% were managers (mostly group leaders). Table 1 gives descriptive summary statistics (mean ± sd) of solidarity and independent variables of solidarity. No differences were found in employee solidarity as reported by managers and other professionals (*t* = 1·2; *P* = 0·218).

**Table 1:** Descriptive statistics of the variables used in the regression analyses (*N *= 313).

	*N*	Mean	sd	Min	Max
Solidarity	298	3·36	0·51	1·90	4·80
Centralization	307	3·03	1·22	1·00	6·50
Hierarchical culture	306	26·43	15·29	0·00	86·00
Communication					
Formal exchange of information	300	4·19	1·06	1·17	7·00
Informal exchange of information	305	4·91	1·26	1·00	5·00
Leadership					
Transformational	299	3·56	0·67	1·17	5·00
Transactional	302	3·18	0·63	1·20	5·00
Passive	304	2·42	0·69	1·00	4·75

Correlations between solidarity and organizational characteristics are presented in Table 2. All organizational characteristics were significantly correlated with solidarity in the study sample (all at *P* ≤ 0·001). These results indicate that centralization, a hierarchical culture and passive leadership style are negatively associated with solidarity, whereas exchange of information and transactional and transformational leadership styles are positively related.

**Table 2:** Correlations between independent variables and solidarity (*N *= 313).

	*r*	*P*	*N*
Centralization	−0·390	≤0·001	295
Hierarchical culture	−0·360	≤0·001	295
Communication			
Formal exchange of information	0·437	≤0·001	289
Informal exchange of information	0·384	≤0·001	295
Leadership			
Transformational	0·413	≤0·001	290
Transactional	0·235	≤0·001	290
Passive	−0·285	≤0·001	294

Table 3 presents the influence of organizational characteristics on solidarity in long-term care organizations as calculated through multiple regression analysis. A hierarchical culture appears to be negatively associated with employee solidarity (β−0·143; *P* ≤ 0·01). Also, centralization is negatively associated with employee solidarity (β−0·158; *P* ≤ 0·01). We found statistically significant regression coefficients for both formal and informal exchange of information (both *P* < 0·05). Transformational leadership appears to be important for solidarity (β 0·162; *P* < 0·05), but not transactional and passive leadership styles.

**Table 3:** Multiple regression analysis of organizational characteristics on solidarity (*N *= 313).

	Beta
Centralization	−0·158**
Hierarchical culture	−0·143**
Communication	
Formal exchange of information	0·141*
Informal exchange of information	0·129*
Leadership	
Transformational	0·140*
Transactional	0·083
Passive	−0·057
Adjusted *R*² for equation	0·318

* *P *≤ 0·05

** *P *≤ 0·01.

## Discussion

### Study limitations

Our research is not without limitations. The cross-sectional design hampered our ability to capture organizational dynamics and draw causal inferences. Although our study established important associations, it was not possible to determine their direction. Future longitudinal research is necessary to increase our understanding of how solidarity is enhanced and whether or not it actually improves outcomes, i.e., effectiveness and efficiency.

### Employee solidarity in long-term care

The nature of work in long-term care is different from other industries as it is characterized by high levels of interdependence (Gittell *et al.* 2000, Kaissi *et al.* 2003). If nurses, managers and other professionals do not work well together, this can negatively affect the quality of patient care and services (Kvarnstrom 2008). Enhancing solidarity is expected to improve collaboration among them and is seen as an important success factor to improve quality of care. Solidarity is a relatively new field of study in the healthcare setting. Employee solidarity is both necessary and fragile. Nurses, managers and other professionals have challenging tasks in turbulent healthcare systems making it particularly important to understand which organizational characteristics shape their solidarity.

The results with regard to leadership style show that transformational leadership especially is conducive to solidarity among nurses, managers and other professionals. Although transactional and passive leadership styles are associated with solidarity, the transformative style is most supportive and remains important in the the multivariate regression analysis. As noted by Avolio and Bass (1999), transformational leadership can and should be observed at all organizational levels. Based on the evidence, leadership that goes beyond the traditional transactional style to one that is more intellectually stimulating, inspirational and charismatic will probably result in higher levels of solidarity, cohesion, commitment, trust, motivation and performance in organizational environments with high levels of interdependencies (Bass 1990, Avolio & Bass 1999). This notion is further supported by the negative influence of a hierarchical culture and centralization. Solidarity among nurses, managers and other professionals seems to improve when authority is delegated to lower levels of the organizational hierarchy.

With respect to communication, both formal and informal exchanges of information enhance solidarity, suggesting that they should be encouraged and correctly balanced. Formal communication takes place through the authoritative channels of the organization established by management and transmits information such as goals, policies and procedures. Messages in this realm follow a chain of command, meaning information flows from managers to subordinates to the next-ranked staff and so on. An example is a company’s newsletter, which gives employees and clients an idea of its goals and visions. It also includes the transfer of information with regard to memoranda, reports, directions and scheduled meetings. The advantages of formal communication are that they help fix responsibility and maintain authority in an organization. Organizations should beware of becoming too formal as it may lead to a hierarchical culture and, as we have shown, negative effects on solidarity. It is important to realize that our study took place among employees in long-term care organizations with considerable autonomy and challenging tasks. There may be distinctive differences in other organizations with routine tasks and less autonomy for employees. It would take a comparative study across sectors to investigate these differences in relation to employee solidarity.

Informal communication, built around the social relationships of members of the organization, does not flow through lines of authority, but arises through personal needs of an organization’s members. A formal working environment always houses an informal communication network. A strict hierarchical web of communication cannot function efficiently on its own. Although informal communication may disrupt the chain of command, good managers need to allow balance between the two channels. Managers who stroll the corridors and adopt a hands-on approach to handling employee queries are good examples of encouraging informal communication. An organization’s lunch area is another setting where relaxed discussions among employees are encouraged. Quality circles, team work and training programmes – all are outside the chain of command and thus fall under the category of informal communication. As with formal communication, an organization should beware of becoming too informal. It can be difficult to fix responsibility about the accuracy of information – highly important in long-term care organizations. Frequent interaction enhances collaboration and solidarity. A good balance of formal and informal communication channels maintains solidarity, responsibility and authority.

## Conclusion

Our study increased the knowledge of solidarity in the long-term care setting. Organizational characteristics that enhance employee solidarity are (1) high levels of both formal and informal information exchange, (2) low-level authority (3) decentralization and (4) transformational leadership styles. Enhancing solidarity among nurses, managers and other professionals is in turn expected to improve organizations’ effectiveness, efficiency and quality of care. Nurses need to be well informed and have the authority to respond to emerging situations. To improve solidarity among nurses, managers and other professionals, authority should be delegated to lower levels of the organizational hierarchy. In addition, it is important that solidarity is present at all levels in the organization. This especially holds true in an organizational environment such as health care with high levels of interdependencies among employees. This underscores the importance of management’s ability to use the language of cooperation and solidarity as a means to get nurses, managers and other professionals to work together to improve the quality of patient care and services.

## Funding

The research was supported by a grant given by the Netherlands Organization for Health Research and Development [ZonMw, grant numbers 53200005, 60-60900-96-005]. The views expressed in the article are those of the authors.

## Conflict of interest

No conflict of interest has been declared by the authors.

## Author contributions

All authors meet at least one of the following criteria (recommended by the ICMJE: http://www.icmje.org/ethical_1author.html) and have agreed on the final version:

- substantial contributions to conception and design, acquisition of data, or analysis and interpretation of data;
- drafting the article or revising it critically for important intellectual content.

**What is already known about this topic**

- Employee solidarity reportedly improves organizations’ effectiveness and efficiency.
- Nurses, managers and other professionals have challenging tasks in turbulent healthcare systems, making it particularly important to understand which organizational characteristics shape their solidarity.
- Centralization, hierarchical culture, formal and informal exchange of information and leadership style are organizational characteristics that influence solidarity.

**What this paper adds**

- Solidarity is seen as an important success factor in organizations, but it is a relatively new field of study among nurses, managers and other professionals in the long-term care setting.
- This study identifies that the organizational characteristics culture, centralization, exchange of information and leadership style explain employee solidarity in the long-term care sector.
- High levels of both formal and informal information exchange, low-level authority, decentralization and transformational leadership styles positively affect solidarity among nurses, managers and other professionals in the long-term care sector.

**Implications for practice and/or policy**

- Transformational leadership styles (intellectually stimulating, inspirational and charismatic) result in higher levels of solidarity and should therefore be used at all organizational levels.
- To improve solidarity among nurses, managers and other professionals, authority should be delegated to lower levels of the organizational hierarchy.
- With respect to communication, both formal and informal exchanges of information enhance solidarity, suggesting that they should be encouraged and correctly balanced.

## Appendix

### Appendices

Solidarity scale (totally disagree/disagree/nor agree or disagree/agree/totally agree)

- everyone helps when something needs to be done
- in our team people do not take each others’ interests in consideration
- in our team we help each other when there is a need for it
- in our team people do not stick to their word
- in our team people apologize when something turns out wrong
- in our team people do not show up when something needs to be done
- in our team no one tries to profit at the costs of others
- in our team you should not ask others for help
- in our team people meet their commitments, even if it is not always convenient
- if people do something that’s annoying to others they don’t care
